# Standard Selection Treatments with Sulfadiazine Limit Plasmodium yoelii Host-to-Vector Transmission

**DOI:** 10.1128/msphere.00106-22

**Published:** 2022-05-19

**Authors:** Kelly T. Rios, Taylor M. Dickson, Scott E. Lindner

**Affiliations:** a Department of Biochemistry and Molecular Biology, The Huck Center for Malaria Research, Pennsylvania State University, University Park, Pennsylvania, USA; The Hebrew University of Jerusalem

**Keywords:** *Plasmodium*, gametocyte, sulfadiazine, malaria, malaria transmission, *Plasmodium yoelii*, rodent malaria parasite

## Abstract

Some antimalarial drugs that have lost clinical usefulness have been repurposed for experimental applications. One example is sulfadiazine, an analog of *p*-aminobenzoic acid (pABA), which inhibits the parasite’s folate synthesis pathway to block DNA synthesis. Sulfadiazine treatment of mice infected with Plasmodium yoelii and P. berghei is routinely used to enrich for gametocytes by killing asexual blood-stage parasites, but it is not well known if there are downstream effects on transmission. To determine if there was a significant effect of sulfadiazine exposure upon transmission, we transmitted Plasmodium yoelii (17XNL strain) parasites to Anopheles stephensi mosquitoes and evaluated the prevalence and intensity of infection under different sulfadiazine treatment conditions. We observed that there was a reduction in both the number of mosquitoes that became infected and in the intensity of infection if parasites were exposed to sulfadiazine in the mouse host or mosquito vector. Sulfadiazine treatment could be marginally overcome if mosquitoes were provided fresh pABA. In contrast, we determined that gametocytes exposed to sulfadiazine could develop into morphologically mature ookinetes *in vitro*, thus sulfadiazine exposure in the host may be reversible if the drug is washed out and the parasites are supplemented with pABA in the culture media. Overall, this indicates that sulfadiazine dampens host-to-vector transmission and that this inhibition can only be partially overcome by exposure to fresh pABA *in vivo* and *in vitro*. Because gametocytes are of great interest for developing transmission-blocking interventions, we recommend the use of less disruptive approaches for gametocyte enrichment.

**IMPORTANCE** In this work, we have uncovered a substantial problem with how many studies of the sexual stages of rodent malaria parasites are conducted. Briefly, the isolation of sexual blood-stage *Plasmodium* parasites, or gametocytes, is essential to study pretransmission and transmission-stage biology of malaria. A routine method for the isolation of this specific stage in rodent-infectious malaria models is drug treatment with sulfadiazine, an antifolate that selectively kills actively replicating asexual blood-stage parasites but not gametocytes. Thus, researchers use this as a convenient way to produce highly enriched gametocyte samples. However, in this work, we describe how this standard drug selection with sulfadiazine not only kills asexual blood-stage parasites but also substantially impacts host-to-vector transmission.

## INTRODUCTION

Sulfa drugs have been used to treat human-infectious Plasmodium falciparum; sulfadoxine, in combination with another antifolate, pyrimethamine, had been used extensively as an antimalarial therapy in areas where malaria is endemic (1). The widespread emergence of resistant parasites after drug pressure in clinical samples and in *in vitro* cultures makes these antifolates inappropriate for antimalarial monotherapies (2–4). Because of this, sulfadoxine-pyrimethamine is now provided with artesunate as a WHO-recommended first-line combination therapy for the treatment of P. falciparum malaria in the WHO SE Asia regions (5).

Although sulfa drugs may not be as effective for treating human malaria infections today, sulfadiazine has been adapted as a commonly used tool to study rodent-infectious malaria parasites as it selectively kills the actively replicating asexual blood stages of the parasite and effectively enriches for sexual-stage gametocytes (6). Sulfa drugs act as antifolates by competitively inhibiting the interaction of an essential enzyme in the *de novo* folate synthesis pathway, dihydropteroate synthase (DHPS), with its substrate, *p*-aminobenzoic acid (pABA). Antifolate drugs are effective against *Plasmodium* as they cannot use preformed folates like their hosts can and thus require *de novo* folate synthesis for the downstream generation of nucleic acids for DNA replication (7, 8). As such, actively replicating parasites, like those in asexual blood stages, are killed by sulfadiazine exposure, while nonreplicating gametocytes survive and may be enriched by this treatment. DNA replication is essential not only in the asexual blood stages of the parasite but also in early mosquito stages, including male gametogenesis, genome replication in the fertilized zygote to form a tetraploid ookinete, and rounds of DNA replication in the oocyst. *Plasmodium* parasites mainly source pABA from their hosts, although *de novo* pABA synthesis in P. berghei was recently observed when pABA in the rodent host diet was restricted (9). In agreement with this, earlier work reported that pABA-deficient diets in rodent hosts are responsible for poor parasite growth and infection, indicating that pABA is an essential host-derived nutrient (10, 11). Indeed, newborn mice on naturally pABA-deficient milk diets suppressed parasitemia with Py17XNL infection and removal of pABA from the rodent diet reduced parasite load (12). Therefore, it is notable that pABA is present in normal laboratory mouse feed at levels that allow for asexual blood stages to progress without additional supplementation (~175 μg/kg of body weight in conventional mouse feed) (9). Similarly, laboratory-reared mosquitoes are commonly supplemented with pABA (0.05%, wt/vol) in their sugar water to enhance oocyst numbers in transmission experiments (13).

Commonly, treatment with sulfadiazine to enrich for P. berghei or P. yoelii sexual blood-stage parasites is accomplished by providing 10 to 30 mg/liter (30 to 120 μM) sulfadiazine in the rodent host’s drinking water for 24 to 48 h prior to parasite collection (14). Beetsma and colleagues established the use of sulfadiazine treatment to enrich for gametocytes from rodent blood-stage infection, observing a reduction in asexual blood-stage parasitemia with 10 mg/liter sulfadiazine supplemented in the host drinking water for 2 days, with no effect on gametocytemia (6). When Anopheles gambiae mosquitoes were allowed to feed on untreated or sulfadiazine-treated PbANKA-infected mice, there was no difference in the intensity or prevalence of midgut oocyst infection. From this, they concluded that sulfadiazine is an effective treatment to isolate large numbers of gametocytes and that these gametocytes are transmissible to mosquitoes (6). Although Beetsma and colleagues did not report defects in PbANKA transmission, early mosquito stages could still be impacted by sulfadiazine treatment, as they are known to undergo DNA replication in early mosquito development during gametogenesis, zygote-to-ookinete maturation, and in later development during sporogony (15). *Plasmodium* transmission studies starting from the 1940s have indicated that there is an effect of sulfadiazine exposure on host-to-vector transmission, although this early work used a variety of mosquito vectors and *Plasmodium* species combinations and was limited in the number of mosquitoes tested and their analyses of transmission (13, 16–20). Perhaps because of this, these studies showed some inconsistencies. For example, work on P. gallinaceum transmission to Aedes aegypti showed that sulfadiazine inhibited sporozoite development (16, 17), but follow-up work a few years later suggested that sulfadiazine or sulfanilamide in the Aedes aegypti diet can even increase the insect’s susceptibility to P. gallinaceum oocyst development (18). Work around the same time implied that P. gallinaceum oocyst growth in *Anopheles quadrimaculatus* is inhibited by sulfadiazine (19), but P. vivax transmission to Anopheles stephensi was not found to be inhibited by sulfadiazine (20). Finally, work on P. berghei NK65 strain parasites in Anopheles stephensi showed that sulfadoxine exposure reduced the number of oocysts in a dose-dependent manner (13). Together these early experiments all pointed in the same direction: that sulfadiazine exposure is detrimental for proper transmission of *Plasmodium* parasites in mosquitoes. However, the limitations of these experiments leave many important details unanswered.

Here, we have investigated the effects of sulfadiazine treatment of mice and mosquitoes upon the transmission of P. yoelii gametocytes to A. stephensi mosquitoes. Specifically, we considered if the timing of exposure to sulfadiazine affects transmission, if pABA can help overcome treatment with sulfadiazine, and if treated parasites will mature *in vitro* as expected. We found that sulfadiazine exposure in the host or mosquito vector resulted in significantly decreased prevalence and intensity of infection in the mosquito midgut. Furthermore, we observed that providing excess pABA to mosquitoes in their sugar water only marginally rescued the effects of sulfadiazine exposure in the host. Finally, when sulfadiazine-treated parasites were cultured *in vitro* to produce ookinetes, no difference in the proportion of mature ookinetes was observed when sulfadiazine was washed out, indicating that the effects of sulfadiazine exposure are reversible.

## RESULTS

### Sulfadiazine treatment of the host limits transmission to mosquitoes.

**FIG 1:**
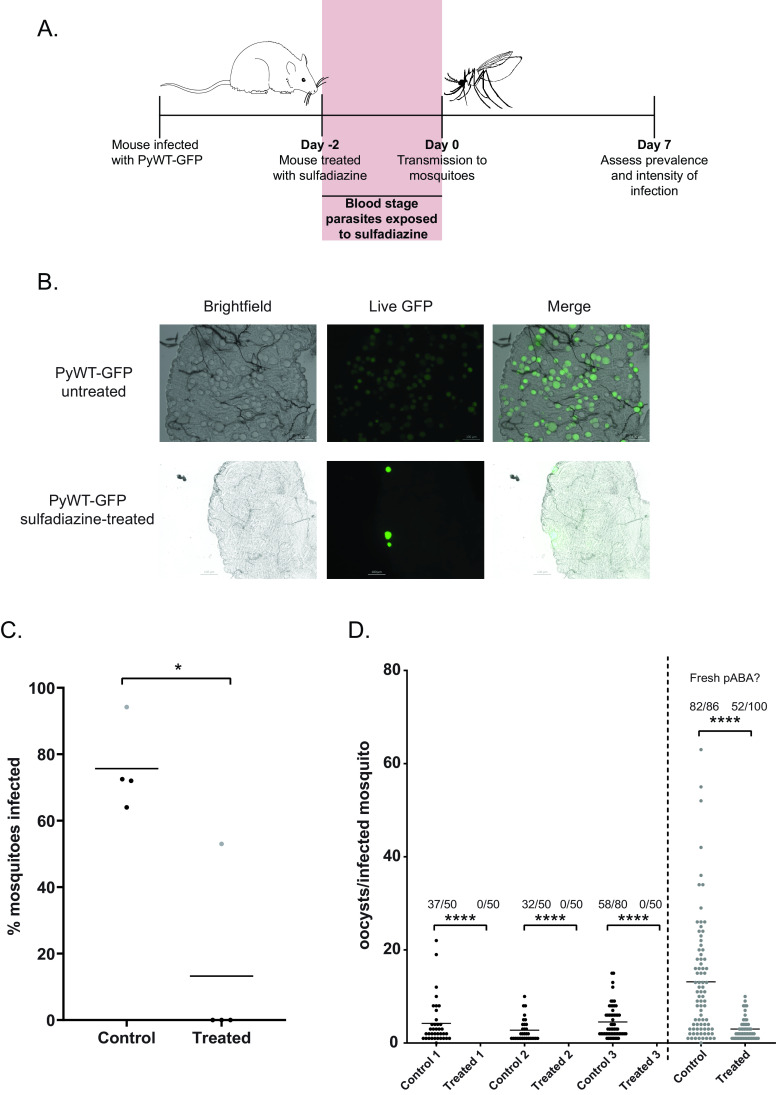
Sulfadiazine exposure in the host limits transmission to the mosquito vector. (A) Mice infected with PyWT-GFP parasites were given standard drinking water (control) or water supplemented with 10 mg/liter sulfadiazine (treated) for 2 days before mosquitoes were allowed to take an infectious blood meal. On day 7 after blood meal, live fluorescence microscopy of dissected mosquito midguts (B) was used to assess the percentage of mosquitoes infected (prevalence) (C) and the number of oocysts per infected mosquito (intensity of infection) (D). Shown are representative images of WT-GFP oocyst-infected midguts dissected 7 days after blood meal from mosquitoes fed on untreated mice (top) or sulfadiazine-treated mice (bottom). Scale bar, 100 μm. (C) The average prevalence of infection for each biological replicate is represented by a data point, and the mean percentage of infected mosquitoes of all replicates is provided as a horizontal line. The gray data points correspond to the fourth replicate in panel D. Mann-Whitney unpaired nonparametric test was used for statistical analyses. *, *P* < 0.05. (D) The intensity of infection as measured by the number of counted oocysts per infected mosquito is plotted, and the number of infected mosquitoes over the total number of mosquitoes counted for each sample is listed above each sample. Biological conditions for the final replicate (gray data points) may have been different, including supplementation of mosquitoes with fresher pABA (tested in Fig. 2). Mann-Whitney unpaired nonparametric test was used for statistical analyses. ****, *P* < 0.0001.

To first test if the standard treatment of infected mice with sulfadiazine for the selection of gametocytes had any effect on parasite transmission, mice were provided with standard drinking water or sulfadiazine-treated drinking water for 2 days leading up to the infectious blood meal to mosquitoes (schematic in Fig. 1A). Because DNA replication in male gametocytes is essential for the production of microgametes in the mosquito, we assessed if male gametocyte activation was affected by sulfadiazine exposure in the rodent host. We detected no defect in male gametogenesis, as assessed by counting exflagellation centers by bright-field microscopy of wet mounts from sulfadiazine-treated and untreated mice (data not shown). Following transmission to A. stephensi mosquitoes, we assessed the prevalence and intensity of infection under each condition 7 days later using live fluorescence microscopy. We did not observe differences in the size or morphology of oocysts that did form in mosquitoes that fed on sulfadiazine-treated mice (Fig. 1B). Parasites treated in the host with sulfadiazine were severely impacted in their ability to transmit to mosquitoes; in three of four biological replicates, no transmission was observed when mosquitoes fed on sulfadiazine-treated mice (Fig. 1C). In the fourth biological replicate (gray data points), mosquitoes that fed on sulfadiazine-treated mice were able to be infected, although at a significantly reduced intensity of infection, with significantly fewer oocysts per midgut observed (Mann-Whitney unpaired nonparametric test, *P* < 0.0001) (Fig. 1D).

### pABA supplementation of mosquitoes does not overcome exposure of parasites to sulfadiazine in the host.

**FIG 2:**
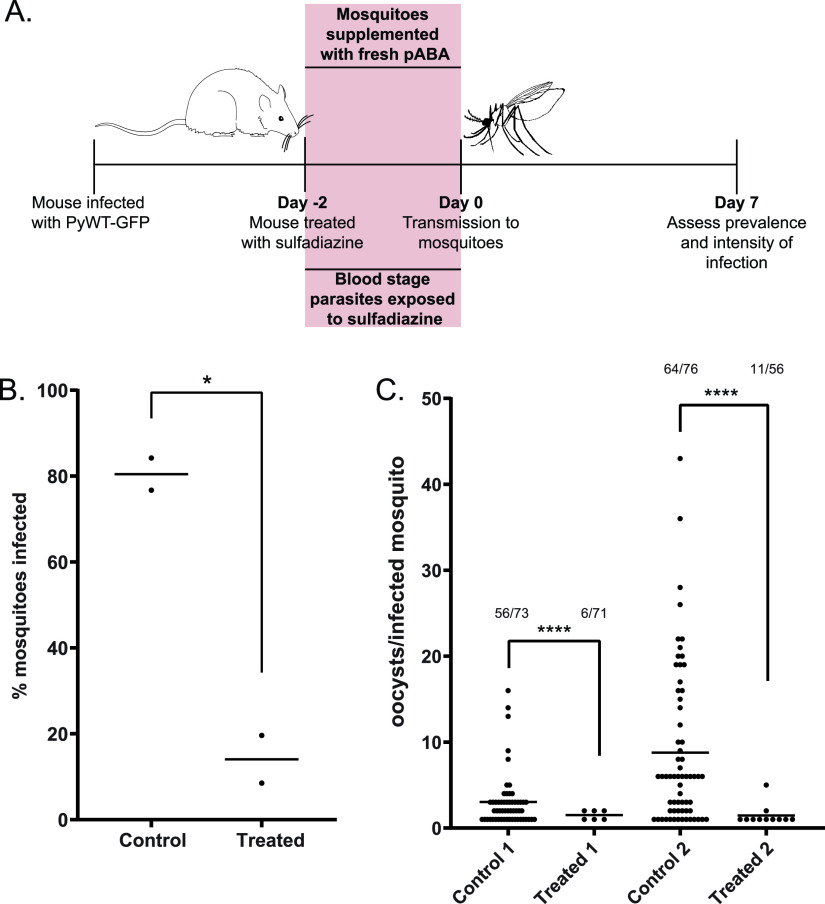
Fresh pABA supplementation can partially recover sulfadiazine exposure of parasites in the host. (A) Mice infected with PyWT-GFP parasites were given standard drinking water (control), or water supplemented with 10 mg/liter sulfadiazine (treated), 2 days before mosquitoes were allowed to take an infectious blood meal. Mosquito sugar water was supplemented daily with freshly diluted pABA (0.05%, wt/vol) to test if fresh pABA can compete with sulfadiazine in the blood meal to recover parasite infection in the mosquito. On day seven after blood meal, the percentage of infected mosquitoes (prevalence) (B) and the number of oocysts per infected mosquito (intensity of infection) (C) were assessed by live fluorescence microscopy. (B) The average prevalence of infection for each biological replicate is represented by a data point, and the mean percentage of infected mosquitoes of all replicates is provided as a horizontal line. Mann-Whitney unpaired nonparametric test was used for statistical analyses. *, *P* = 0.01. (C) The intensity of infection, as measured by the number of counted oocysts per infected mosquito, is plotted. The number of infected mosquitoes out of the total number of mosquitoes counted for each sample is listed above each sample. Mann-Whitney unpaired nonparametric test was used for statistical analyses. ****, *P* < 0.0001.

Because the fourth biological replicate (Fig. 1C and D, gray data points) did result in limited transmission to mosquitoes, we considered whether there may have been different experimental conditions that allowed transmission of sulfadiazine-exposed parasites in this replicate. One such condition that may affect sulfadiazine treatment is the amount of pABA present in the mosquito vector, as sulfadiazine is a structural analog of pABA that acts as a competitive inhibitor of DHPS, and pABA supplementation of mosquitoes allows for increased numbers of oocysts to develop (13). To determine if provision of fresh pABA to the mosquitoes could enable such transmission to occur, we replaced the pABA-supplemented sugar water daily using freshly dissolved pABA both before and after infectious blood meals taken from either control or sulfadiazine-treated mice. Consistent with this, we observed that fresh pABA supplementation enabled parasites to partially overcome exposure to sulfadiazine in the host and to successfully transmit to mosquitoes, albeit still at significantly lower levels than the untreated control (Fig. 2B) (Mann-Whitney unpaired nonparametric test, *P* = 0.01). Moreover, the transmission intensity (oocysts per infected mosquito) was still significantly lower for mosquitoes that fed on treated mice than untreated mice (Fig. 2C) (Mann-Whitney unpaired nonparametric test, *P* < 0.0001). Additionally, fresh pABA supplementation also improved the percentage of mosquitoes infected that fed on control mice compared with routine pABA supplementation (compare Fig. 1C and Fig. 2B) (average percent infected, 75.68% versus 80.45%). This indicates that the standard practice of sulfadiazine treatment of parasites in the rodent host to select for gametocytes is detrimental to parasite transmission and that this practice may introduce unwanted artifacts that could complicate the study of these important transmission-stage parasites.

### Pretreatment of mosquitoes with sulfadiazine reduces the intensity of mosquito infection.

**FIG 3:**
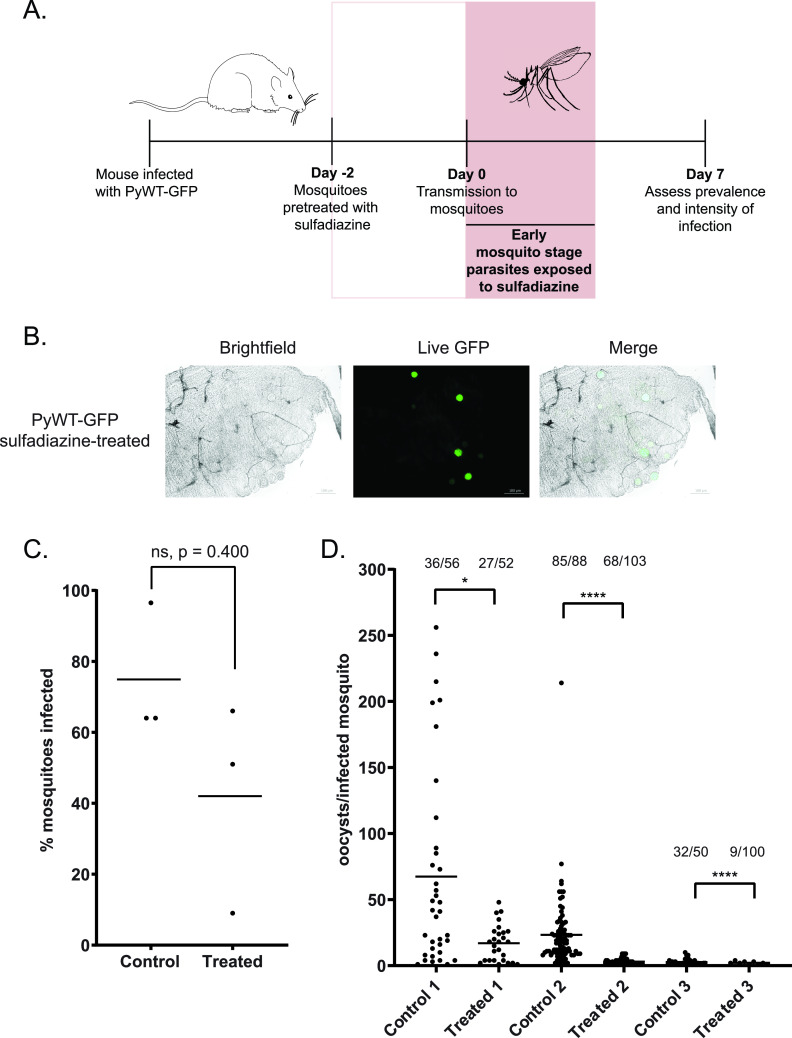
Sulfadiazine exposure to early mosquito stages decreases oocyst intensity. (A) Mosquitoes were given standard sugar-pABA water or sugar-pABA water supplemented with 10 mg/liter sulfadiazine for 2 days leading up to an infectious blood meal from PyWT-GFP-infected mice, such that early mosquito stages are then exposed to sulfadiazine as they are taken up by the mosquito. On day 7 after blood meal, live fluorescence microscopy (B) was used to assess the percentage of mosquitoes infected (prevalence) (C) and the number of oocysts per infected mosquitoes (intensity of infection) (D). (B) Representative images of a WT-GFP oocyst-infected midgut dissected 7 days after blood meal from mosquitoes supplemented with sulfadiazine. Scale bar, 100 μm. (C) The percentage of mosquitoes infected that fed on PyWT-GFP mice when mosquitoes were exposed to sulfadiazine (treated), or not (control), daily leading up to the infectious bloodmeal is plotted. The average percent infection for each biological replicate is represented by each data point, and the mean prevalence of all replicates is provided as a horizontal line. Mann-Whitney unpaired nonparametric test was used for statistical analyses; ns, no significant difference; *P* > 0.05. (D) The intensity of infection, as measured by the number of counted oocysts per infected mosquito, is plotted. The number of infected mosquitoes out of the total number of mosquitoes counted for each sample is listed above each sample. Mann-Whitney unpaired nonparametric test was used for statistical analyses. *, *P* < 0.05; ****, *P* < 0.0001.

As sulfadiazine is bioavailable in the blood of the host, it will also be taken up along with the parasites during a blood meal. This would effectively extend the sulfadiazine exposure to the earliest mosquito stages as well. To test if parasites exposed only to sulfadiazine in the mosquito would have similar effects on transmission, we provided the mosquitoes with sulfadiazine in their sugar-pABA water for 2 days ahead of an infectious blood meal taken from untreated, infected mice (Fig. 3A). We did not observe differences in oocyst size or morphology in treated versus untreated mosquitoes (Fig. 1B and 3B) and did not find any statistically significant effect upon the prevalence of parasite transmission due to sulfadiazine exposure that was restricted to the mosquito midgut (Fig. 3C) (Mann-Whitney unpaired nonparametric test, *P* = 0.400, not significant). Despite this, there was a significant reduction in the number of oocysts per infected mosquito observed when mosquitoes were treated with sulfadiazine (Fig. 3D), indicating that sulfadiazine can affect the early mosquito stages (gametes, zygotes, and ookinetes) as well as the sexual blood-stage gametocytes.

### Sulfadiazine exposure in the host does not affect morphological development of *in vitro* ookinetes.

**FIG 4:**
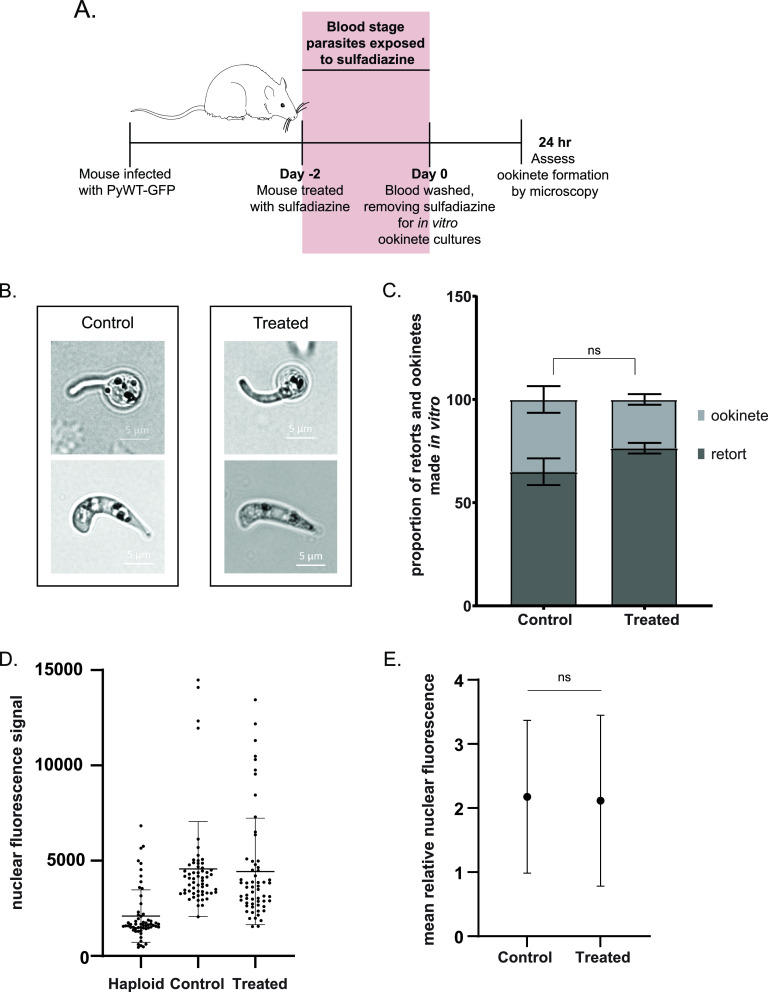
Sulfadiazine exposure to parasites in the host does not affect their ability to morphologically mature *in vitro*. (A) Mice infected with PyWT-GFP parasites were given standard drinking water (control) or drinking water supplemented with 10 mg/liter sulfadiazine (treated) for 2 days before parasite collection by exsanguination. Collected parasites were enriched by a discontinuous Accudenz gradient before being resuspended in sulfadiazine-free ookinete culture medium at room temperature. After 24 h, the proportion of immature retorts and mature ookinetes was assessed by morphology. (B) Representative images of retorts (top) or mature ookinetes (bottom) from control and treated parasites. (C) The proportion of immature retorts and mature ookinetes observed in culture from blood of mice that was supplemented with sulfadiazine (treated) or not (control). A two-proportion Z-score test was used for statistical analysis. ns, no significant statistical difference. *P* > 0.05. Between 115 and 140 cells were counted per sample per biological replicate. (D) Nuclear fluorescence signal was assessed by microscopy of DAPI-stained early asexual blood-stage parasites and mature *in vitro* cultured ookinetes that developed from treated and untreated gametocytes (*n* = 59 cells imaged per sample). Fluorescence signal was calculated by the multiplication of the nuclear area and the average pixel intensity for each imaged cell. The calculated fluorescence intensity for each measured cell is represented by each data point, and the mean and standard deviation for each sample is represented by the horizontal lines. (E) The nuclear fluorescence of mature cultured ookinetes that developed from treated and untreated gametocytes was normalized to the nuclear signal of early asexual blood-stage parasites. The mean normalized fluorescence for each sample is represented by the data points, and the standard deviations are represented by the vertical lines. An unpaired *t* test was used for statistical analysis. ns, no significant statistical difference. *P* > 0.05.

Because sulfadiazine treatment of the rodent host limited parasite development *in vivo* (Fig. 1 and 2) and early mosquito stages were affected by sulfadiazine exposure in the mosquito midgut (Fig. 3), we tested if sulfadiazine treatment had a reversible effect upon parasite development through these stages. To this end, mixed blood-stage parasites from untreated or sulfadiazine-treated mice were collected using an Accudenz gradient and then resuspended and cultured *in vitro* in a defined medium containing pABA (1.0 mg/liter; 7.299 μM) to assess fertilization and ookinete maturation (Fig. 4A). Using differential interference contrast (DIC) microscopy, we did not observe any gross morphological differences in retorts or ookinetes that formed from either sulfadiazine-treated or untreated parasites (Fig. 4B). Quantification of retort and ookinete-stage parasites revealed no statistically significant differences in the proportions of retorts and ookinetes present in culture (Fig. 4C) (two-proportion Z-score test, *P* > 0.05, not significant). We were unable to assess *in vivo* ookinetes from the infected mosquito blood bolus by microscopy because of technical challenges in detection due to autofluorescence of the robust mosquito microbiome after blood meal. Because DNA replication occurs as the fertilized zygote matures into a tetraploid ookinete, we attempted to measure the difference in nucleic acid staining with DRAQ5 from 2N (zygote) to 4N (ookinete) by flow cytometry. However, we were unable to resolve the shift in DNA content from 2N to 4N between stages by this method, which is consistent with published use of flow cytometry to measure DNA content between unfertilized female gametes (1N) and formed zygotes (2N/4N) but which was not sufficiently robust to quantify the increase from 2N to 4N due to the heterogeneity in the timing of this process (21). Because flow-cytometric analysis of nucleic acid content of ookinetes was inconclusive, we used fluorescence microscopy to quantify the amount of DAPI staining present in parasites derived from untreated or sulfadiazine-treated mice and then subjected them to *in vitro* ookinete cultures (Fig. 4D). We did not observe statistically significant differences in the average DAPI signal between control and treated parasites when normalized to that of haploid (1N) asexual blood-stage parasites (Fig. 4E). The high variance in the observed DAPI signal may represent the biological asynchronicity of ookinete maturation and DNA replication. We therefore conclude that prior exposure of gametocytes to sulfadiazine in the mouse does not inhibit DNA replication or ookinete development *in vitro* when sulfadiazine is washed out.

Taken together, these data demonstrate that treatment with sulfadiazine impacts not only asexual blood-stage parasites but also gametocytes and early mosquito-stage parasites. This indicates that not only does the exposure of gametocytes to sulfadiazine affect transmission but also that exposure of the early mosquito stages to sulfadiazine within the mosquito midgut has a transmission-blocking effect as well. Moreover, it is feasible that sulfadiazine can be introduced to the mosquito midgut via the rodent host or directly by the mosquito vector. Finally, consistent with sulfa drugs being competitive inhibitors of *Plasmodium* DHPS, we also conclude that the effects of sulfadiazine treatment upon *Plasmodium* development in early mosquito stage is reversible and can be at least partially overcome by competition by pABA supplementation of mosquitoes.

## DISCUSSION

Sulfadiazine treatment is routinely used in rodent-infectious *Plasmodium* research labs to select for sexual blood-stage gametocytes, the only life stage transmissible from host to vector. The enrichment of this stage from other blood-stage parasites is therefore critical to be able to robustly study the biology of host-to-vector transmission (epigenetic studies, transcriptomic studies, etc.). In particular, the development of novel transmission-blocking strategies to prevent the further spread of malaria is dependent on a deep understanding of sexual-stage biology. However, the underappreciated effect of gametocyte enrichment by sulfadiazine could be impacting these studies of parasite transmission.

Here, we have shown that parasite transmission is impaired by sulfadiazine exposure of parasites in both the host and in the mosquito vector. The timing of exposure to sulfadiazine treatment is important and impactful, as mosquitoes that fed on sulfadiazine-treated mice likely have taken up sulfadiazine with their blood meal. In this scenario, if sulfadiazine was present in the blood bolus, it is feasible that early mosquito-stage zygotes and ookinetes were exposed to the drug as well. Because sulfonamides are pABA analogs, we tested if treatment of mosquitoes with fresh pABA water could overcome sulfadiazine exposure. Ultimately, pABA supplementation of sulfadiazine-treated parasites resulted in only a partial restoration of transmission capability, as there were still significantly lower numbers of mosquitoes infected and lower numbers of oocysts per infected mosquito with sulfadiazine-treated parasites, even upon fresh pABA supplementation. *In vitro*, sulfadiazine-exposed gametocytes can still develop into morphologically mature ookinetes with no measurable difference in the extent of DNA replication occurring compared to untreated parasites. The blood used for *in vitro* culturing of ookinetes was enriched using an Accudenz gradient to collect infected red blood cells, so effectively any sulfadiazine in the whole blood collected from the mice was washed out. The excess pABA present in the ookinete culture media can then outcompete for binding to DHPS and allow the parasites to develop as expected. It is likely that ookinetes that remain exposed to sulfadiazine in *in vitro* culture would not develop properly due to inhibition of DNA replication, as early mosquito-stage parasites exposed to sulfadiazine in the mosquito midgut do not progress through the life cycle as untreated parasites do. That male gametocytes can activate and undergo gametogenesis, as observed by bright-field microscopy, and that fertilization can occur *in vitro* when sulfadiazine is removed indicates that DNA replication in male gametocytes is not substantially affected by sulfadiazine exposure. This is supported by Delves *et al.* (22), in which the authors assessed the effects of various antimalarial compounds on P. falciparum 3D7 male and female gametocyte activation. No significant difference in male gametogenesis was observed when cultures were treated with 1 μM sulfadiazine for 24 h before activation was assayed (22). This leads us to conclude that exposure within the mosquito vector is responsible for the transmission defects we have observed.

It is possible that sulfadiazine treatment of the mouse or of mosquitoes before transmission affects the mosquito midgut microbiome as well as the *Plasmodium* parasites. We did not observe adverse effects on mosquito survival with sulfadiazine supplementation. Although we have not directly studied the effects of sulfadiazine treatment on the mosquito midgut microbiome here, when placed in the context of previous studies, it is most plausible that the sulfadiazine-induced transmission defect we observed is parasite specific. Several studies that have explored these effects are worth noting. First, it was demonstrated that the mosquito midgut bacteria can have a negative effect on *Plasmodium* development in the mosquito and that antibiotic treatment leads to higher parasite infection (23). Additionally, it was shown that mosquitoes that fed on *Plasmodium*-infected blood containing penicillin and streptomycin had enhanced mosquito-stage infections and reduced bacterial growth (24). This antibiotic treatment perhaps gives the parasites a competitive advantage over the bacterial flora for nutrients in the mosquito midgut. If sulfadiazine treatment was adversely affecting the mosquito microbiome, we could similarly anticipate that the parasites would have enhanced development in the mosquitoes rather than a defect such as what we have observed.

Recent work exposing mosquitoes to antimalarials, rather than antibiotics, may prove to be a new way to prevent new infections in natural transmission settings. For example, Anopheles gambiae exposed to atovaquone before an infectious blood meal results in a mosquito-stage infection deficiency (25). Testing more antimalarial drugs in this fashion could improve transmission-blocking strategies for the elimination of malaria. This is another example of antimalarials taking on a new life after blood-stage parasite resistance has emerged.

Finally, there are other means to enrich for gametocytes that may be preferable and less disruptive to transmission, like flow cytometry using gametocyte-specific antibodies or available fluorescent reporter lines using male- or female-enriched promoters (26, 27) or magnetic enrichment (28, 29). Based on these results, we strongly encourage their use over sulfadiazine selection to produce as minimally perturbed parasites as possible for the study of host-to-vector transmission.

## MATERIALS AND METHODS

### Ethics statement.

All vertebrate animal care followed the Association for Assessment and Accreditation of Laboratory Animal Care (AAALAC) guidelines and was approved by the Pennsylvania State University Institutional Animal Care and Use Committee (IACUC number PRAMS201342678). All procedures involving vertebrate animals were conducted in strict accordance with the recommendations in the *Guide for the Care and Use of Laboratory Animals* of the National Institutes of Health (30) with approved Office for Laboratory Animal Welfare (OLAW) assurance.

### Use and maintenance of experimental animals.

Six- to 8-week-old female Swiss Webster (SW) mice were used for all experiments in this work. Anopheles stephensi mosquitoes were reared and maintained at 24°C and 70% humidity under 12-h light/dark cycles and were fed 0.05%, wt/vol, pABA (number 100536-250G; Sigma-Aldrich) supplemented into 10%, wt/vol, sugar water. Mice were infected intraperitoneally with PyWT-GFP transgenic parasites that constitutively express green fluorescent protein (GFP) under a constitutive EF1 alpha promoter from the *pyp230p* dispensable locus (described previously [31]).

### Treatment of mice and mosquitoes with sulfadiazine.

Mice were provided with standard drinking water before infection. After infection with PyWT-GFP parasites, the mice were kept on standard drinking water or were provided water supplemented with 10 mg/liter sulfadiazine (number AAA12370-30; VWR) for 2 days before the infectious blood meal to select for gametocytes. Mosquitoes were provided normal pABA-sugar water (0.05%, wt/vol, pABA, 10%, wt/vol, sugar) during rearing and kept on normal pABA-sugar water or supplied with pABA-sugar water supplemented with 10 mg/liter sulfadiazine for 2 days before the infectious blood meal. After the blood meal, mosquitoes were again provided standard sugar-pABA drinking water for the duration of the *Plasmodium* infection.

### Host-to-vector transmission of Plasmodium yoelii.

Mice infected with PyWT-GFP parasites were screened daily for parasitemia by Giemsa-stained thin blood smears and for the presence of male gametogenesis (visible as discrete exflagellation centers) via wet mount of a drop of blood incubated at room temperature for 8 to 10 min, as described previously (32). On the day of peak exflagellation, the infected mice were anesthetized with a ketamine-xylazine cocktail and the mosquitoes were allowed to feed on the mice once for 15 min. Mosquito midguts were dissected 7 days after blood meal, and the prevalence and intensity of infection were assessed by differential interference contrast (DIC) and live fluorescence microscopy (Zeiss Axioscope A1 with 8-bit AxioCam ICc1 camera) using a 100× oil objective and processed by Zen 2012 (blue edition) imaging software.

### Production of *in vitro* ookinetes.

Mice infected with PyWT-GFP parasites were supplied with standard drinking water or were treated with 10 mg/liter sulfadiazine-treated drinking water to select for gametocytes for 2 days leading up to exsanguination. Parasitemia was assessed by Giemsa-stained thin blood smears, and centers of movement were assessed to establish optimal experimental timing as described above. Blood was collected by cardiac puncture and then was maintained at 37°C in incomplete RPMI with 25 mM HEPES and l-glutamine (number 45000-412; VWR). Infected red blood cells were enriched by an Accudenz discontinuous gradient as previously described (33). Ookinete cultures were generated as previously described, with modifications for P. yoelii (34, 35). Briefly, the Accudenz-collected cells were added to *in vitro* ookinete medium (RPMI 1640, 20%, vol/vol, fetal bovine serum [number MT35011CV; Fisher Scientific], 0.05%, wt/vol, hypoxanthine [number AC122010250; Fisher Scientific], 100 μM xanthurenic acid [number D120804-1G; Sigma-Aldrich], pH 8.2, at 22°C) and were allowed to develop for 24 h at room temperature. The pH was adjusted to pH 8.2, using KOH, rather than pH 7.5 for P. berghei, and cultures were maintained for 24 h at ambient temperature (22°C) rather than in a 19°C incubator. Retorts and ookinetes were then observed and quantified by DIC and fluorescence microscopy (Zeiss Axioscope A1 with 8-bit AxioCam ICc1 camera) using the 100× oil objective and processed by Zen 2012 (blue edition) imaging software.

### Measurement of DNA content in treated/untreated parasites via *in vitro* ookinete culture.

The DNA content of *in vitro* cultured mature ookinetes was measured by microscopy as previously described (36, 37). Early asexual blood-stage parasites (rings, early trophozoites), cultured control, or sulfadiazine-treated ookinetes were stained with 4′,6-diamidino-2-phenylindole (DAPI) (number D9564; Sigma-Aldrich) and imaged by fluorescence microscopy as described above (Zeiss Axioscope A1 with 8-bit AxioCam ICc1 camera) using a 100× oil objective and processed by Zen 2012 (blue edition) imaging software. The nuclear area and pixel intensity for imaged cells were measured in ImageJ, and the nuclear fluorescence intensity was calculated as nuclear area (pixels) × mean fluorescence intensity (relative units) (36–38). The nuclear fluorescence intensity was standardized to the DNA content of asexual blood-stage rings and early trophozoites (36, 37).

### Statistical analyses.

Statistical differences in midgut oocyst infection numbers and numbers of infected mosquitoes were analyzed by Mann-Whitney unpaired nonparametric test, with a *P* value of <0.05 indicating statistical significance. *P* values are listed where significant. The Mann-Whitney nonparametric test was used because the data do not follow a normal distribution and control and experimental groups are independent of each other. A two-proportion Z-score test was used for statistical analyses of ookinete and retort formation *in vitro* under no treatment conditions or with sulfadiazine treatment; a *P* value of <0.05 indicates significance. An unpaired *t* test was used to compare the mean fluorescence intensities of mature ookinetes that developed from control and sulfadiazine-treated gametocytes; a *P* value of <0.05 was used as a threshold of statistical significance. All statistical analyses were performed using GraphPad Prism (v8).

### Data availability.

All data related to this study are provided in the manuscript.

## References

[1] Ekland EH, Fidock DA. 2008. In vitro evaluations of antimalarial drugs and their relevance to clinical outcomes. Int J Parasitol 38:743–747. doi:10.1016/j.ijpara.2008.03.004. 18406409 PMC2430068

[2] Triglia T, Menting JG, Wilson C, Cowman AF. 1997. Mutations in dihydropteroate synthase are responsible for sulfone and sulfonamide resistance in Plasmodium falciparum. Proc Natl Acad Sci USA 94:13944–13949. doi:10.1073/pnas.94.25.13944. 9391132 PMC28412

[3] Wang P, Read M, Sims PF, Hyde JE. 1997. Sulfadoxine resistance in the human malaria parasite Plasmodium falciparum is determined by mutations in dihydropteroate synthetase and an additional factor associated with folate utilization. Mol Microbiol 23:979–986. doi:10.1046/j.1365-2958.1997.2821646.x. 9076734

[4] Wang P, Lee C-S, Bayoumi R, Djimde A, Doumbo O, Swedberg G, Dao LD, Mshinda H, Tanner M, Watkins WM, Sims PFG, Hyde JE. 1997. Resistance to antifolates in Plasmodium falciparum monitored by sequence analysis of dihydropteroate synthetase and dihydrofolate reductase alleles in a large number of field samples of diverse origins. Mol Biochem Parasitol 89:161–177. doi:10.1016/S0166-6851(97)00114-X. 9364963

[5] World Health Organization. 2020. World malaria report 2020. https://www.who.int/publications/i/item/9789240015791.

[6] Beetsma AL, van de Wiel TJ, Sauerwein RW, Eling WM. 1998. Plasmodium berghei ANKA: purification of large numbers of infectious gametocytes. Exp Parasitol 88:69–72. doi:10.1006/expr.1998.4203. 9501851

[7] Triglia T, Cowman AF. 1999. The mechanism of resistance to sulfa drugs in Plasmodium falciparum. Drug Resist Updat 2:15–19. doi:10.1054/drup.1998.0060. 11504465

[8] Müller IB, Hyde JE. 2013. Folate metabolism in human malaria parasites–75 years on. Mol Biochem Parasitol 188:63–77. doi:10.1016/j.molbiopara.2013.02.008. 23500968

[9] Matz JM, Watanabe M, Falade M, Tohge T, Hoefgen R, Matuschewski K. 2019. Plasmodium para-aminobenzoate synthesis and salvage resolve avoidance of folate competition and adaptation to host diet. Cell Rep 26:356–363. doi:10.1016/j.celrep.2018.12.062. 30625318

[10] Maegraith BG, Deegan T, Jones ES. 1952. Suppression of malaria (P berghei) by milk. Br Med J 2:1382–1384. doi:10.1136/bmj.2.4799.1382. 12997791 PMC2022317

[11] Kicska GA, Ting L-M, Schramm VL, Kim K. 2003. Effect of dietary p-aminobenzoic acid on murine Plasmodium yoelii infection. J Infect Dis 188:1776–1781. doi:10.1086/379373. 14639551

[12] Parra M, Yang J, Weitner M, Akkoyunlu M. 2021. Neonatal mice resist Plasmodium yoelii infection until exposed to para-aminobenzoic acid containing diet after weaning. Sci Rep 11:90. doi:10.1038/s41598-020-79703-2. 33420157 PMC7794322

[13] Peters W, Ramkaran AE. 1980. The chemotherapy of rodent malaria. XXXII. The influence of p-aminobenzoic acid on the transmission of Plasmodium yoelii and P berghei by Anopheles stephensi. Ann Trop Med Parasitol 74:275–282. doi:10.1080/00034983.1980.11687345. 6994664

[14] Doolan DL (ed). 2002. Malaria methods and protocols. Springer Science & Business Media, New York, NY.

[15] Aly ASI, Vaughan AM, Kappe SHI. 2009. Malaria parasite development in the mosquito and infection of the mammalian host. Annu Rev Microbiol 63:195–221. doi:10.1146/annurev.micro.091208.073403. 19575563 PMC2841446

[16] Terzian LA. 1947. A method for screening antimalarial drugs in the mosquito host. J Parasitol 33:15. 20271936

[17] Gerberg EJ. 1971. Evaluation of antimalarial compounds in mosquito test systems. Trans R Soc Trop Med Hyg 65:358–363. doi:10.1016/0035-9203(71)90014-9. 4397766

[18] Terzian LA. 1950. The sulfonamides as factors in increasing susceptibility to parasitic invasion. J Infect Dis 87:285–290. doi:10.1093/infdis/87.3.285. 14794969

[19] Terzian LA, Stahler N, Ward PA. 1952. The effect of antibiotics and metabolites on the immunity of mosquitoes to malarial infection. J Infect Dis 90:116–130. doi:10.1093/infdis/90.2.116. 14917887

[20] Terzian LA, Stahler N, Dawkins Jr AT. 1968. The sporogonous of Plasmodium vivax in Anopheles mosquitoes as a system for evaluating the prophylactic and curative capabilities of potential antimalarial compounds. Exp Parasitol 23:56–66. doi:10.1016/0014-4894(68)90042-8. 4876903

[21] Vega-Rodriguez J, Perez-Barreto D, Ruiz-Reyes A, Jacobs-Lorena M. 2015. Targeting molecular interactions essential for Plasmodium sexual reproduction. Cell Microbiol 17:1594–1604. doi:10.1111/cmi.12458. 25944054 PMC4668941

[22] Delves MJ, Ruecker A, Straschil U, Lelièvre J, Marques S, López-Barragán MJ, Herreros E, Sinden RE. 2013. Male and female Plasmodium falciparum mature gametocytes show different responses to antimalarial drugs. Antimicrob Agents Chemother 57:3268–3274. doi:10.1128/AAC.00325-13. 23629698 PMC3697345

[23] Smith RC, Vega-Rodríguez J, Jacobs-Lorena M. 2014. The Plasmodium bottleneck: malaria parasite losses in the mosquito vector. Mem Inst Oswaldo Cruz 109:644–661. doi:10.1590/0074-0276130597. 25185005 PMC4156458

[24] Gendrin M, Rodgers FH, Yerbanga RS, Ouédraogo JS, Basáñez M-G, Cohuet A, Christophides GK. 2015. Antibiotics in ingested human blood affect the mosquito microbiota and capacity to transmit malaria. Nat Commun 6; 6:5921. doi:10.1038/ncomms6921. PMC4338536 25562286

[25] Paton DG, Childs LM, Itoe MA, Holmdahl IE, Buckee CO, Catteruccia F. 2019. Exposing Anopheles mosquitoes to antimalarials blocks Plasmodium parasite transmission. Nature 567:239–243. doi:10.1038/s41586-019-0973-1. 30814727 PMC6438179

[26] Ponzi M, Sidén-Kiamos I, Bertuccini L, Currà C, Kroeze H, Camarda G, Pace T, Franke-Fayard B, Laurentino EC, Louis C, Waters AP, Janse CJ, Alano P. 2009. Egress of Plasmodium berghei gametes from their host erythrocyte is mediated by the MDV-1/PEG3 protein. Cell Microbiol 11:1272–1288. doi:10.1111/j.1462-5822.2009.01331.x. 19438517

[27] Bowman LM, Finger LE, Hart KJ, Lindner SE. 2020. Definition of constitutive and stage-enriched promoters in the rodent malaria parasite, Plasmodium yoelii. Malar J 19:424. doi:10.1186/s12936-020-03498-w. 33228734 PMC7685602

[28] Fivelman QL, McRobert L, Sharp S, Taylor CJ, Saeed M, Swales CA, Sutherland CJ, Baker DA. 2007. Improved synchronous production of Plasmodium falciparum gametocytes in vitro. Mol Biochem Parasitol 154:119–123. doi:10.1016/j.molbiopara.2007.04.008. 17521751

[29] Ribaut C, Berry A, Chevalley S, Reybier K, Morlais I, Parzy D, Nepveu F, Benoit-Vical F, Valentin A. 2008. Concentration and purification by magnetic separation of the erythrocytic stages of all human Plasmodium species. Malar J 7:45. doi:10.1186/1475-2875-7-45. 18321384 PMC2292734

[30] National Research Council. 2011. Guide for the care and use of laboratory animals, 8th ed. National Academies Press, Washington, DC.

[31] Muñoz EE, Hart KJ, Walker MP, Kennedy MF, Shipley MM, Lindner SE. 2017. ALBA4 modulates its stage-specific interactions and specific mRNA fates during Plasmodium yoelii growth and transmission. Mol Microbiol 106:266–284. doi:10.1111/mmi.13762. 28787542 PMC5688949

[32] Hart KJ, Oberstaller J, Walker MP, Minns AM, Kennedy MF, Padykula I, Adams JH, Lindner SE. 2019. Plasmodium male gametocyte development and transmission are critically regulated by the two putative deadenylases of the CAF1/CCR4/NOT complex. PLoS Pathog 15:e1007164. doi:10.1371/journal.ppat.1007164. 30703164 PMC6355032

[33] Moll K, et al. 2013. Methods in malaria research, 6th ed. BEI Resources, Manassas, VA.

[34] Modrzynska K, Pfander C, Chappell L, Yu L, Suarez C, Dundas K, Gomes AR, Goulding D, Rayner JC, Choudhary J, Billker O. 2017. A knockout screen of ApiAP2 genes reveals networks of interacting transcriptional regulators controlling the Plasmodium life cycle. Cell Host Microbe 21:11–22. doi:10.1016/j.chom.2016.12.003. 28081440 PMC5241200

[35] Janse CJ, Mons B, Rouwenhorst RJ, van der Klooster PF, Overdulve JP, van der Kaay HJ. 1985. In vitro formation of ookinetes and functional maturity of Plasmodium berghei gametocytes. Parasitology 91:19–29. doi:10.1017/S0031182000056481. 2863802

[36] Bushell ESC, Ecker A, Schlegelmilch T, Goulding D, Dougan G, Sinden RE, Christophides GK, Kafatos FC, Vlachou D. 2009. Paternal effect of the nuclear formin-like protein MISFIT on Plasmodium development in the mosquito vector. PLoS Pathog 5:e1000539. doi:10.1371/journal.ppat.1000539. 19662167 PMC2715856

[37] Reininger L, Billker O, Tewari R, Mukhopadhyay A, Fennell C, Dorin-Semblat D, Doerig C, Goldring D, Harmse L, Ranford-Cartwright L, Packer J, Doerig C. 2005. A NIMA-related protein kinase is essential for completion of the sexual cycle of malaria parasites. J Biol Chem 280:31957–31964. doi:10.1074/jbc.M504523200. 15970588

[38] Schindelin J, Arganda-Carreras I, Frise E, Kaynig V, Longair M, Pietzsch T, Preibisch S, Rueden C, Saalfeld S, Schmid B, Tinevez J-Y, White DJ, Hartenstein V, Eliceiri K, Tomancak P, Cardona A. 2012. Fiji: an open-source platform for biological-image analysis. Nat Methods 9:676–682. doi:10.1038/nmeth.2019. 22743772 PMC3855844

